# Characterising those with incident polymyalgia rheumatica in primary care: results from the PMR Cohort Study

**DOI:** 10.1186/s13075-016-1097-8

**Published:** 2016-09-07

**Authors:** Sara Muller, Samantha L. Hider, Toby Helliwell, Sarah Lawton, Kevin Barraclough, Bhaskar Dasgupta, Irena Zwierska, Christian D. Mallen

**Affiliations:** 1 Arthritis Research UK Primary Centre, Research Institute for Primary Care & Health Sciences, Keele University, Keele, Staffordshire ST5 5BG UK; 2 Haywood Rheumatology Centre, Stoke on Trent, UK; 3 Painswick Surgery, Gloucestershire, UK; 4 Southend University Hospital, Southend, Essex UK

**Keywords:** Polymyalgia rheumatica, Epidemiology, Disease activity

## Abstract

**Background:**

The aim was to characterise the sociodemographic, general health and polymyalgia rheumatica (PMR)-specific features of participants in a large inception cohort of patients with PMR diagnosed in UK primary care.

**Methods:**

Patients (*n* = 739) with a new diagnosis of PMR were referred into the study and mailed a questionnaire detailing their general health and sociodemographic characteristics in addition to the symptoms of and treatment for PMR. Characteristics of responders and non-responders were compared and descriptive statistics were used to characterise the health of the cohort.

**Results:**

A total of 654 individuals responded to the questionnaire (adjusted response 90.1 %). Responders and non-responders were similar in age, gender and deprivation (based on postcode). The mean (standard deviation) age of the recruited cohort was 72.4 (9.3) years; 62.2 % were female. The sample reported high levels of pain and stiffness (8 out of 10 on numerical rating scales) and reported stiffness that lasted throughout the day. High levels of functional impairment, fatigue, insomnia and polypharmacy were also reported. Overall, women reported worse general and PMR-specific health than did men.

**Conclusions:**

This first primary care cohort of patients with incident PMR is similar in demographic terms to cohorts recruited in secondary care. However, the extent of symptoms, particularly reported stiffness, is higher than has been described previously. Given the majority of patients with PMR are exclusively managed in primary care, this cohort provides important information on the course of PMR in the community that will help clinicians managing this painful and disabling condition.

## Background

Polymyalgia rheumatica (PMR) is a common inflammatory rheumatic condition in those over the age of 50, with an estimated lifetime prevalence of 2.4 % in women and 1.7 % in men [1]. Classic presentation involves rapid onset of bilateral pain and stiffness in the shoulders and hip girdle, usually in combination with raised inflammatory markers. Although common, it has received relatively little research attention, particularly in the primary care setting, where the majority of patients are diagnosed and managed [2, 3].

To date, our understanding of the epidemiology of PMR is not as advanced as for other inflammatory rheumatological conditions. Those diagnosed with PMR are more likely to be female and have an average age at diagnosis of approximately 72 years [4]. PMR is more prevalent at more northerly latitudes [5, 6] and is frequently associated with giant cell arteritis (GCA) with between 16 and 21 % of patients with PMR having evidence of GCA and up to 60 % of patients with GCA having symptoms of PMR [7]. Recent studies have identified increased risk of vascular disease [8] and cancer diagnosis [9] following a diagnosis of PMR, though the mechanisms behind these associations remain uncertain.

The PMR Cohort Study was established to investigate the primary care epidemiology of PMR. In this inception cohort of general practitioner (GP)-diagnosed PMR, patients are followed via postal surveys and medical records for two years post-PMR diagnosis. Medical records are also available for the two years prior to diagnosis. This paper presents data from the baseline survey phase of the study, describing the recruited sample in terms of sociodemographic data, general health and disease-specific characteristics.

## Methods

### Study design

The PMR Cohort Study is an inception cohort of people diagnosed with PMR in general practice. The methods of the study have been described in detail elsewhere [10]. Briefly, participants were referred from 382 general practices across England between June 2012 and June 2014. When seeing a potentially eligible patient, an electronic prompt alerted the GP to the study as specific disease codes were entered into the patient record, reminding them of the patients’ eligibility and the British Society for Rheumatology (BSR) guidelines for diagnosing PMR. In practices without electronic prompts in place, laminated cards with these details were placed, as an aide memoire, in consulting rooms.

Study packs, including written copies of the BSR PMR clinical guidelines were provided for each practice. General practitioners (GPs) referred patients who provided verbal consent to receive more information into the study via secure fax or email when they made a new diagnosis of PMR (no previous Read-coded consultation in the last three years). Participants were then sent a baseline questionnaire. Participants who did not respond within three weeks were sent a reminder questionnaire. Response to the baseline questionnaire implied consent to be followed up via postal survey at six further time points over two years (1, 4, 8, 12, 18 and 24 months). Participants were also asked whether they consented to the linkage of their survey data to their primary care medical records and nationally held registers. This study describes data from the baseline phase of the study only.

### Baseline data collection

The baseline questionnaire collected information on PMR symptoms at the time of diagnosis, treatments received for PMR, general health, lifestyle, function, sociodemographics and participants’ views on the causes of their PMR.

#### Sociodemographic information

The date of birth (used to calculate age), gender and postcode (used to calculate indices of multiple deprivation [11] of all patients referred into the study by their GP were available from the referral form. The baseline questionnaire also collected information on current employment status (employed, retired, unemployed, housewife/husband, sick or other), ethnicity (white, Asian/Asian British, black/African/Caribbean/African/black British, mixed or other), socioeconomic class based on current or last job title (“High managerial, administrative and professional”, “Intermediate” or “Routine and manual”, according to the Office for National Statistics criteria [12], whether the participant lived alone or not and marital status (married, separated, divorced, widowed, cohabiting or single).

#### General health, lifestyle and function measures

General health-related quality of life was assessed using the descriptive component of the Euroqol-5D (EQ-5D) [13]. The five items of this questionnaire are combined to create an index where 1 is considered perfect health and 0 is considered to be representative of death or unconsciousness. The worst health state on this index is valued at -0.59 (i.e. worse than death).

Function in activities of daily living in the past week was assessed using the modified Health Assessment Questionnaire (mHAQ) [14, 15]. Scores on this 8-item instrument range from 0 to 3, with a score of less than 0.3 considered to represent normal functioning [16].

Fatigue in the past week was assessed using the 13-item Functional Assessment of Chronic Illness Therapy (FACIT)-fatigue questionnaire that has been shown to be valid and reliable for use with patients with systemic lupus erythematosus. Scores on the questionnaire range from 0 to 52 and higher scores indicate less fatigue. In the general US population aged 18 years and over, the mean score on this instrument has been shown to be 40.1 (SD 10.4) [17].

Insomnia in the previous two weeks was assessed using the 7-item Insomnia Severity Index (ISI), with individuals classified as having no clinically significant insomnia, subthreshold insomnia, clinical insomnia (moderate severity) or clinical insomnia (severe).

Anxiety and depression in the previous two weeks were assessed using the 7-item Generalised Anxiety Disorder (GAD) questionnaire [18] and the 8-item Patient Health Questionnaire [19], respectively. Anxiety was classified as none (score 0–4), mild (5–9), moderate (10–14) or severe (15–21). Depression was classified as none (score 0–4), mild (5–9), moderate (10–14), moderately severe (15–19) or severe (20–24). Participants were also asked whether they had someone they could rely on for emotional and practical support.

Smoking status (never smoked, ex-smoker or current smoker) and frequency of alcohol consumption (daily or almost daily, 3–4 times a week, 1–2 times a week, 1–3 times a month, special occasions or never) were reported by responders, along with height and weight, which were used to calculate body mass index (BMI).

#### Symptoms of and treatments for PMR

Participants reported the levels of pain and stiffness they were experiencing when their GP diagnosed them with PMR on a 0 to 10 scale where 0 was no pain/stiffness and 10 was “pain as bad as can be”/“very severe” stiffness, by circling the number corresponding to the level of pain/stiffness as appropriate. They were also asked to check boxes indicating which times of day they experienced stiffness (none, morning, lunchtime, afternoon, early evening, late evening or during the night), the duration of any morning stiffness (none, 1–15 minutes, 16–45 minutes, 46–60 minutes or >1 hour) and whether they could raise their arms above their head when they were diagnosed with PMR. Participants were also asked to report whether they used prednisolone or a range of other medications for their PMR.

### Statistical analyses

Responders were compared to non-responders by age, gender and tertile of deprivation score using basic descriptive statistics. The sociodemographic data, general health and lifestyle and PMR-specific symptoms and treatments of the sample were described using means and standard deviations (SD), medians and interquartile ranges (IQR) and frequencies and percentages, as appropriate. The characteristics of the sample were compared between men and women using the chi-square, rank sum or *t* test as applicable. Levels of pain and stiffness were compared graphically and by calculating correlation coefficients.

## Results

### Response to the survey

There were 739 people referred into the study. Three of these were duplicate patients, two had died, four had incorrect address information, and four were otherwise ineligible. This left an eligible sample of 726, of whom 654 responded to the baseline questionnaire (adjusted response rate 90.1 %). The age and gender of responders (mean (standard deviation) age 72.4 years (9.3), 62.2 % female) and non-responders (mean (standard deviation) age 72.6 years (10.0), 62.5 % female) were similar overall, although non responders were more likely to be in the highest tertile of deprivation (32.3 % responders vs 38.9 % non-responders).

### Sociodemographic characteristics

The majority of the sample were retired (*n* = 513, 79.9 %), with only 77 (11.9 %) reporting themselves to be employed. Almost all of the sample identified themselves as being of white ethnicity (*n* = 640, 98.2 %). Approximately a third of the sample had or had previously held higher managerial, administrative or professional occupations, 28 % had intermediate occupations and 40 % had routine occupations. The majority of people reported that they did not live alone (*n* = 467, 71.6 %) and 412 (63.4 %) reported that they were married, and 143 (22 %) were widowed.

### General health and lifestyle characteristics

The median EQ-5D descriptive component score was 0.73 (IQR 0.59, 0.85) and was higher in men than in women (Table 1). The median mHAQ score was 0.40 (IQR 0.0, 1.0), with a higher score in women than in men (0.5 (0.0, 1.0) vs 0.38 (0.0, 0.9); *p* = 0.0434). The mean FACIT-Fatigue score was 33.9 (SD 12.4), with women reporting being more fatigued than men (32.0 (12.8) vs 37.0 (10.8); *p* < 0.0001). Approximately 24 % of the sample were considered to have clinical insomnia, 13 % anxiety (moderate or severe) and 22 % depression (moderate, moderately severe or severe). The prevalence of all of these symptoms was higher in women than in men (*p* < 0.0001).

**Table 1:** General health characteristics of responders

	All	Female	Male	*P* value
EQ-5D descriptive component score^a^ (median, IQR)	0.73 (0.59, 0.85)	0.69 (0.52,0.81)	0.76 (0.62, 1.0)	0.0008
mHAQ (median, IQR)	0.40 (0.0, 1.0)	0.5 (0.0, 1.0)	0.38 (0.0, 0.9)	0.0434
FACIT-Fatigue (mean, SD)^a^	33.9 (12.4)	32.0 (12.9)	37.0 (10.8)	<0.0001
Insomnia (ISI) (*n*, %)				0.003
No clinically significant insomnia	244 (39.6)	130 (33.9)	114 (48.9)	
Subthreshold insomnia	227 (36.9)	151 (39.4)	76 (32.6)	
Clinical insomnia (moderate severity)	112 (18.2)	78 (20.4)	34 (14.6)	
Clinical insomnia (severe)	33 (5.4)	24 (6.3)	9 (3.9)	
Anxiety (GAD–7) (*n*, %)				<0.0001
None (0–4)	397 (65.0)	220 (58.4)	177 (75.6)	
Mild (5–9)	134 (21.9)	93 (24.7)	41 (17.5)	
Moderate (10–14)	49 (8.0)	41 (10.9)	8 (3.4)	
Severe (15–21)	31 (5.1)	23 (6.1)	8 (3.4)	
Depression (PHQ-8) (*n*, %)				<0.0001
None (0–4)	318 (52.7)	171 (46.3)	147 (62.8)	
Mild (5–9)	153 (25.4)	93 (25.2)	60 (25.6)	
Moderate (10–14)	71 (11.8)	56 (15.2)	15 (6.4)	
Moderately severe (15–19)	38 (6.3)	30 (8.1)	8 (3.4)	
Severe (20–24)	23 (3.8)	19 (5.2)	4 (1.7)	
Smoking status (*n*, %)				<0.0001
Never smoked	318 (49.3)	223 (55.9)	95 (38.6)	
Ex-smoker	287 (44.5)	153 (38.4)	134 (54.5)	
Current smoker	40 (6.2)	23 (5.8)	17 (6.9)	
Frequency of alcohol consumption (*n*, %)				<0.0001
Daily or almost daily	82 (12.6)	26 (6.4)	56 (22.8)	
3–4 times a week	82 (12.6)	42 (10.3)	40 (16.3)	
1–2 times a week	113 (17.3)	56 (13.8)	57 (23.2)	
1–3 times a month	80 (12.3)	52 (12.8)	28 (11.4)	
Special occasions	185 (28.4)	141 (34.7)	44 (17.9)	
Never	110 (16.9)	89 (21.9)	21 (8.5)	
Body mass index (*n*, %)				0.001
< 25 kg/m^2^	221 (33.9)	128 (33.2)	83 (35.0)	
25.0–29.9 kg/m^2^	251 (40.3)	139 (36.0)	112 (47.3)	
30.0–34.9 kg/m^2^	100 (16.1)	69 (17.9)	31 (13.1)	
≥ 35 kg/m^2^	61 (9.8)	50 (13.0)	11 (4.6)	
Count on someone to provide emotional support (*n*, %)				0.009
Yes	553 (85.2)	355 (87.7)	198 (81.2)	
No	35 (5.4)	23 (5.7)	12 (4.9)	
No need	61 (9.4)	27 (6.7)	34 (13.9)	
Count on someone to provide practical support (*n*, %)				<0.0001
Yes	519 (79.7)	347 (85.5)	172 (70.2)	
No	32 (4.9)	18 (4.4)	14 (5.7)	
No need	100 (15.4)	41 (10.1)	59 (24.1)	

^a^High score indicates better functioning. *EQ-5D* EuroQoL 5 dimensions, *mHAQ-D* Modified Health Assessment Questionnaire Disability Index, *FACIT* Functional Assessment of Chronic Illness Therapy, *ISI* Insomnia Severity Index, *GAD-7* Generalised Anxiety Disorder 7-item questionnaire, *PHQ-8* 8-item Patient Health Questionnaire

Current smoking was reported by 6.2 % (*n* = 40) of the sample, with 44.5 % reporting previously having smoked. Men were more likely to have smoked previously than women. A quarter of the sample reported drinking three times a week or more, and 45.3 % (*n* = 295) reported never drinking or drinking only on special occasions. More frequent drinking was more common in men than in women.

One third of the sample (*n* = 221, 33.9 %) had self-reported BMI under <25 kg/m^2^ and 161 (25.9 %) had BMI >30 kg/m^2^. Being overweight (BMI ≥25 to <30 kg/m^2^ was more common in men and obesity (BMI ≥30 kg/m^2^) was more common in women).

The majority of people reported having someone to provide emotional (*n* = 553, 85.2 %) or practical support (519, 79.7 %) or not to need this sort of support (emotional: *n* = 61 (9.4 %); practical: *n* = 100 (15.4 %)). Women were more likely to desire and have access to both types of help (for emotional support *p* = 0.009; for practical support *p* < 0.0001).

### Symptoms of PMR

**Fig. 1:**
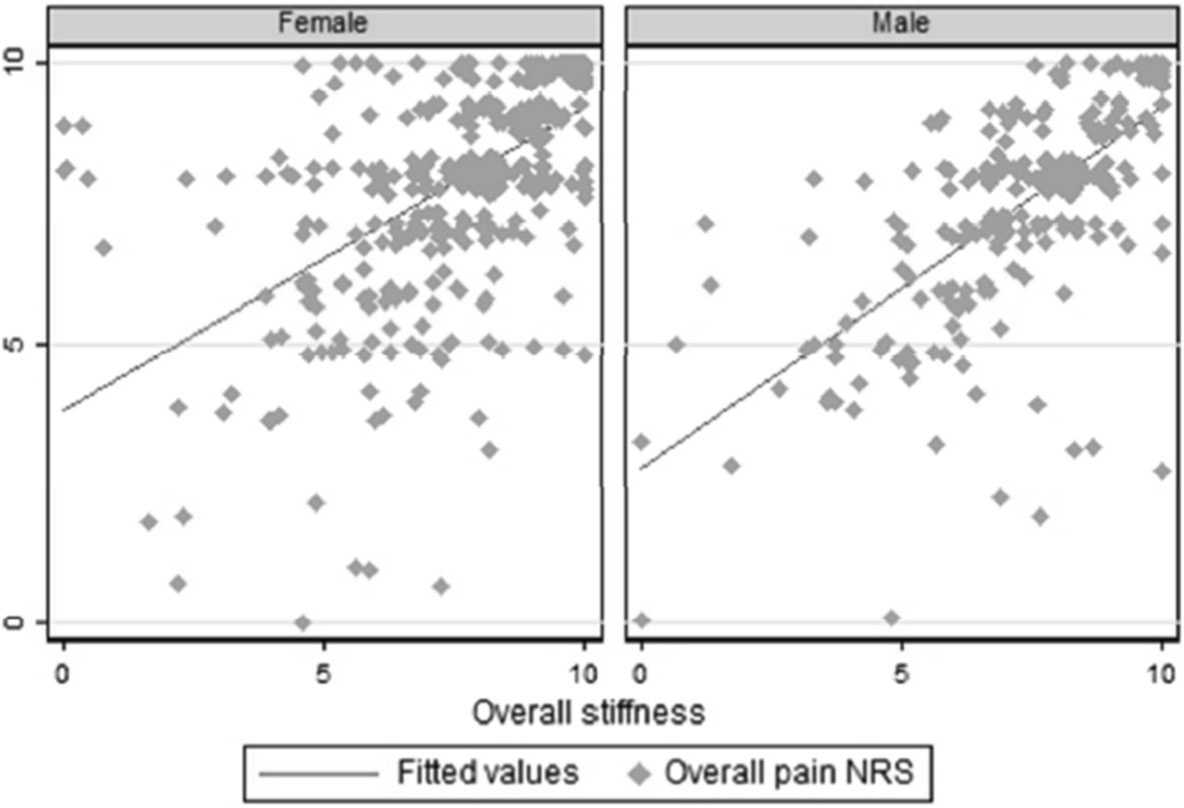
The association between pain and stiffness scores (0–10 numerical rating scales (*NRS*)) by gender

The median (IQR) scores were 8 (7, 9) for pain and 8 (7, 9) for stiffness. In both of these ratings, women reported higher scores than men (Table 2) (for pain *p* = 0.0005; for stiffness *p* = 0.0049). There was strong correlation between pain and stiffness scores in both men (*r* = 0.6671) and women (*ρ* = 0.5766) (Fig. 1).

**Table 2:** Polymyalgia rheumatica (PMR)-related characteristics of responders

	All	Female	Male	*P* value
Median (IQR) pain score (NRS)	8 (7, 9)	8 (7, 10)	8 (7, 9)	0.0005
Median (IQR) stiffness score (NRS)	8 (7, 9)	8 (7, 9)	8 (6, 9)	0.0049
Times of day when stiffness present (*n*, %)^a^				
None	15 (2.3)	10 (2.5)	5 (2.0)	0.724
Morning	619 (95.2)	387 (95.6)	232 (94.7)	0.617
Lunchtime	368 (56.6)	227 (56.1)	141 (57.6)	0.708
Afternoon	357 (54.9)	219 (54.1)	138 (56.3)	0.576
Early evening	394 (60.6)	257 (63.5)	137 (55.9)	0.057
Late evening	446 (68.6)	288 (71.1)	158 (64.5)	0.078
During the night	467 (71.9)	302 (74.6)	165 (67.4)	0.047
Duration of morning stiffness (*n*, %)				0.491
None	12 (1.9)	8 (2.0)	4 (1.7)	
1–15 minutes	31 (4.9)	16 (4.0)	15 (6.3)	
16–45 minutes	69 (10.8)	41 (10.3)	28 (11.7)	
46–60 minutes	72 (11.3)	50 (12.6)	22 (9.2)	
> 1 hour	454 (71.2)	283 (71.1)	171 (71.3)	
Able to raise both arms above head when first saw GP (*n*, %)				0.101
Yes	186 (28.8)	107 (26.6)	79 (32.5)	
No	413 (64.0)	261 (64.9)	152 (62.6)	
Don’t know	46 (7.1)	34 (8.5)	12 (4.9)	
Use of prednisolone (*n*, %)				0.484
Yes	626 (96.9)	390 (96.5)	236 (97.5)	
No	20 (3.1)	14 (3.5)	6 (2.5)	
Other medications for PMR (*n*, %)^a^				
Paracetamol	248 (37.9)	176 (43.2)	72 (29.2)	<0.0001
Paracetamol and codeine	137 (21.0)	96 (23.6)	41 (16.6)	0.033
Non-steroidal anti-inflammatories	69 (10.6)	44 (10.8)	25 (10.1)	0.781
Strong prescription painkillers	86 (13.2)	62 (15.2)	24 (9.7)	0.043
Gastro-protection	332 (50.8)	210 (51.6)	122 (49.4)	0.585
Calcium and vitamin D	304 (46.5)	199 (48.9)	105 (42.5)	0.113
Osteoporosis treatments	170 (26.0)	119 (29.2)	51 (20.7)	0.015
Alternative therapies	26 (4.0)	20 (4.9)	6 (2.4)	0.115
Anti-depressants	55 (8.4)	47 (11.6)	8 (3.2)	<0.0001
Other	100 (15.3)	55 (13.5)	45 (18.2)	0.105

^a^Can sum to >100 %, as respondents were able to choose more than one option. *NRS* numerical rating scales

Almost all participants reported stiffness in the morning (*n* = 619, 95.2 %) with more than half also reporting stiffness at all other times of day. A similar proportion of men and women reported daytime stiffness, with women reporting more stiffness during the night (*p* = 0.047). Duration of morning stiffness was generally reported to be more than an hour (*n* = 454, 71.2 %), with only 17.6 % reporting stiffness in the morning lasting 45 minutes or less. Stiffness duration was similar in men and women. Approximately two thirds of people (*n* = 413, 64 %) reported being unable to raise their arms above their head when they were diagnosed with PMR, and 7.1 % (*n* = 46) reported that they did not know. There was no significant difference between genders in reported ability to do this task (*p* = 0.101).

### Treatments for PMR

Use of prednisolone among survey respondents was almost universal (n = 626, 96.9 %). Use of other medications for PMR was also prevalent, with 37.9 % (*n* = 248) reporting using paracetamol and 21 % (*n* = 137) reporting use of combination analgesia. Both of these medications were more common in women than in men (paracetamol: 43.2 % vs 29.2 %; combination analgesia: 23.6 % vs 16.6 %). Participants also reported using non-steroidal anti-inflammatories (*n* = 69, 10.6 %), strong prescription painkillers (e.g. tramadol) (*n* = 86, 13.2 %) (again this was more common in women (15.2 % vs 9.7 %)) and alternative therapies (e.g. homeopathy, acupuncture, herbal medicine) (*n* = 26, 4.0 %). Patients also attributed use of gastro-protective (n = 332, 50.8 %), osteoporosis (*n* = 170, 26.0 %) and calcium and vitamin D (*n* = 304, 46.5 %) treatments to their PMR: 55 participants (8.4 %) reported taking anti-depressants in relation to their PMR. Use of osteoporosis treatments and anti-depressants were more common in women than in men (osteoporosis treatment: 29.2 % vs 42.5 %; anti-depressants: 11.6 % vs 3.2 %).

## Discussion

Polymyalgia rheumatica is largely managed in primary care [2, 3], yet to date there is a paucity of research evidence from patients managed in this setting. As such, clinicians rely on data generated in secondary care that can be challenging to generalise to patients in the community, who may have less severe disease [20] and different prognostic trajectories. This study is the first to characterise a large primary care sample of PMR patients at the time of their diagnosis, thereby providing an unselected and more representative PMR population.

Participant demographics seen in this inception cohort are similar to those recruited from hospital populations, with an average age at disease onset of approximately 72 years and around two thirds of participants being female. Also in keeping with existing evidence on the propensity of this condition to affect those of Northern European descent, the cohort self-identifies almost entirely as being of white ethnicity, despite having recruited from a large range of geographical areas with differing levels of ethnic diversity. Similarly to a previous population study, there is a broad spread of occupational class across the sample [21]. This suggests that hospital PMR cohorts have similar demographics to this unselected primary care sample, providing some reassurance as to the accuracy of GP diagnosis for participants in this cohort. However, unlike the classic description of stiffness predominantly in the morning, within this cohort stiffness was severe and diurnal, with more than 60 % also reporting stiffness in the evening and the majority also describing night-time stiffness. In part this may reflect questionnaire design, in that participants were asked specifically about the presence of stiffness at other times of the day, although as qualitative data in both patients with rheumatoid arthritis [22] and PMR [23] highlights that stiffness may be more varied and complex than is usually reported and is present at various times of day, not just in the morning. Given that stiffness is considered key as part of the core domain set for PMR by Outcome Measures in Rheumatology (OMERACT) [24], we felt that timing of stiffness warranted further exploration within the cohort.

The reported high levels of pain and stiffness that persisted throughout the day (especially in women) may explain the high proportion reporting use of pain killers and non-steroidal anti-inflammatory drugs in addition to prednisolone, especially in women. Furthermore, the greater severity of symptoms in women is in concordance with a previous case series from a tertiary referral clinic [25].

Comparison of the current study to those reported in the literature suggests that overall levels of quality of life [26, 27], depression [28, 29] and obesity [28, 29] are similar to those expected in the population of this age, and levels of daily alcohol consumption [29], current smoking [8, 29] and anxiety are lower [28], although the age structure of available comparator populations varies between studies.

Fatigue was greater in the sample than the in general US population [17], and physical functioning was much better in the PMR population (*n* = 85) used to evaluate change in patient-reported outcomes in PMR [30] where the baseline median (IQR) mHAQ scores were much higher (1.1 (0.8, 1.6)) than in this cohort, although the scores at one week (0.4 (0.1, 0.8)) were similar to those in the current study (0.4 (0.0, 1.0)). In part, this may reflect patient recall bias, with patients asked to recall baseline disability or may reflect patients with a more severe spectrum of disease being referred to specialist services; however, reported duration of morning stiffness and oral glucocorticoid use were similar between studies, although this cohort had higher median (IQR) levels of pain (8 (7, 9) on a numerical rating scale) compared to this American College of Rheumatology (ACR)/European League Against Rheumatism (EULAR) sample [30].

Whilst this study has many strengths, including its size and the availability of detailed information, it also has a number of limitations that need to be considered. First, the sample consists of patients with a GP diagnosis of PMR. Diagnosing PMR is notoriously difficult, without a single gold-standard diagnostic test to inform clinicians. The recent ACR/EULAR classification criteria highlight the challenges in diagnosing PMR, with 10 out of 128 people (7.8 %) diagnosed with PMR by specialists subsequently having their diagnosis revised [30]. Although rheumatology specialists may criticise the use of GP diagnosis in this study, the majority of patients with PMR are exclusively managed in the community and as such GPs and rheumatologists are likely to encounter a different spectrum of disease. In this study, we optimised the diagnostic process by providing BSR guidance in the consultation to support decision-making. Furthermore, the rate of referral into the study of around 4.4 patients per 10,000 per year in those aged over 40 years, is similar to or below that previously reported in primary care data (e.g. see [4]) providing further confidence in our cohort.

A further limitation is the reliance on self-reported data, and although questionnaires were mailed to participants as soon as a diagnosis was made, some recall was required. This is necessary for many of the constructs, as they cannot be objectively measured (e.g. pain, stiffness and quality of life), although for others (e.g. medications) this is not necessarily the case. However, additional information on the patient’s attribution of their medication to PMR was possible through this use of self-reported data. At future stages of the study, these data will be linked to primary care consultation and prescription records.

## Conclusions

PMR has generally been considered to be a relatively mild condition, associated with few other health complications. However, these patients report high levels of pain and stiffness, which is prolonged after the morning time, along with high levels of oral steroid use and polypharmacy. It is also clear that women report worse scores than men in almost all aspects of disease activity, functioning and mental health. In addition, they are more likely to report taking a range of medications in addition to glucocorticoids for their PMR, notably strong painkillers and anti-depressants.

Although this is the first study to characterise a cohort of patients with incident PMR in primary care, further work is needed to establish whether this group would all be considered to have PMR if they visited a rheumatologist. Further studies will be required to determine whether those with more depression or poorer physical functioning have a worse prognosis over the course of their PMR.

## Abbreviations

ACR: American College of Rheumatology
BMI: Body mass index
BSR: British Society for Rheumatology
EQ-5D: Euroqol-5D
EULAR: European League Against Rheumatism
FACIT: Functional Assessment of Chronic Illness Therapy
GAD: Generalised Anxiety Disorder
GCA: Giant cell arteritis
GP: General practitioner
ISI: Insomnia Severity Index
mHAQ: Health Assessment Questionnaire
NIHR: National Institute for Health Research
NRS: Numerical rating scales
PMR: Polymyalgia rheumatica
